# The translational paradox of ADAMTS-5 in osteoarthritis: mechanistic centrality but limited clinical efficacy

**DOI:** 10.3389/fmed.2026.1877932

**Published:** 2026-10-28

**Authors:** Yi Li, Jiangzhou Chu, Shugang Qin, Shengbin Liu

**Affiliations:** 1 School of Medical Technology, Urban Vocational College of Sichuan, Chengdu, China; 2 Department of Experimental Research, Sichuan Clinical Research Center for Cancer, Sichuan Cancer Center, Sichuan Cancer Hospital & Institute, Affiliated Cancer Hospital of University of Electronic Science and Technology of China, Chengdu, China; 3 Department of Critical Care Medicine, Frontiers Science Center for Disease-related Molecular Network, State Key Laboratory of Biotherapy and Cancer Center, West China Hospital, Sichuan University, Chengdu, China

**Keywords:** ADAMTS-5, aggrecan degradation, extracellular matrix degradation, inflammatory signaling, osteoarthritis, precision medicine

## Abstract

Osteoarthritis (OA) is a heterogeneous whole-joint disease for which no disease-modifying therapies have demonstrated consistent clinical efficacy. Among matrix-degrading enzymes, ADAMTS-5 has strong genetic and pharmacological support as a major aggrecanase in experimental OA, whereas its quantitative contribution in human disease is more context dependent. This mechanistic rationale positioned ADAMTS-5 as a leading disease-modifying target. However, the clinical record requires careful separation by development stage: the Phase II ROCCELLA trial of GLPG1972/S201086 showed no significant structural or symptomatic benefit, whereas the anti-ADAMTS-5 nanobody M6495 has so far been evaluated mainly for Phase I safety, pharmacokinetics, and pharmacodynamics rather than efficacy. In this review, we re-evaluate ADAMTS-5 within a network integrating inflammatory signaling, mechanotransduction, extracellular-matrix remodeling, and tissue adaptation. We examine how disease stage, protease redundancy, biomarker-outcome dissociation, delivery limitations, and patient heterogeneity can weaken translation from enzyme inhibition to clinical benefit. We further discuss molecular endotyping, single-cell and spatial profiling, and data-driven stratification as tools to identify patients and disease states in which aggrecanase activity is most likely to be therapeutically relevant. Future strategies should therefore emphasize stage-specific and locally controlled intervention, biomarker-informed enrichment, and rational combination therapy rather than uniform systemic inhibition. ADAMTS-5 thus provides a useful model for understanding why a biologically compelling OA target may fail when therapeutic timing, tissue context, and patient selection are not aligned.

## 1 Introduction

Osteoarthritis (OA) is a leading cause of disability worldwide, particularly in aging populations. Its global burden continues to rise with increasing life expectancy, posing substantial socioeconomic and healthcare challenges (1–5). Despite its high prevalence, current treatments remain largely symptomatic and no disease-modifying osteoarthritis drug (DMOAD) has yet demonstrated consistent clinical efficacy (6–8). At the tissue level, OA is characterized by progressive cartilage degeneration, subchondral bone remodeling, synovial inflammation, and periarticular changes. Early extracellular-matrix (ECM) degradation, particularly loss of aggrecan, compromises cartilage biomechanical integrity. ADAMTS-5 is a major aggrecanase in experimental OA and has been strongly implicated in this process, although the relative contribution of ADAMTS-5 and other proteases in human OA varies with tissue context and disease stage (9–15).

Accumulating evidence from genetic and pharmacological studies indicates that ADAMTS-5 plays a central role in OA pathogenesis (Figure 1). Genetic ablation studies have demonstrated that ADAMTS-5-deficient mice are protected from cartilage degradation in multiple OA models (16), while pharmacological inhibition of ADAMTS-5 reduces aggrecan loss and structural damage in preclinical settings (16). These findings have positioned ADAMTS-5 as a promising therapeutic target for disease modification. Consequently, substantial efforts have been devoted to the development of ADAMTS-5 inhibitors, including small molecules (17), monoclonal antibodies (18), and nucleic acid-based approaches such as siRNA.

**Figure 1:**
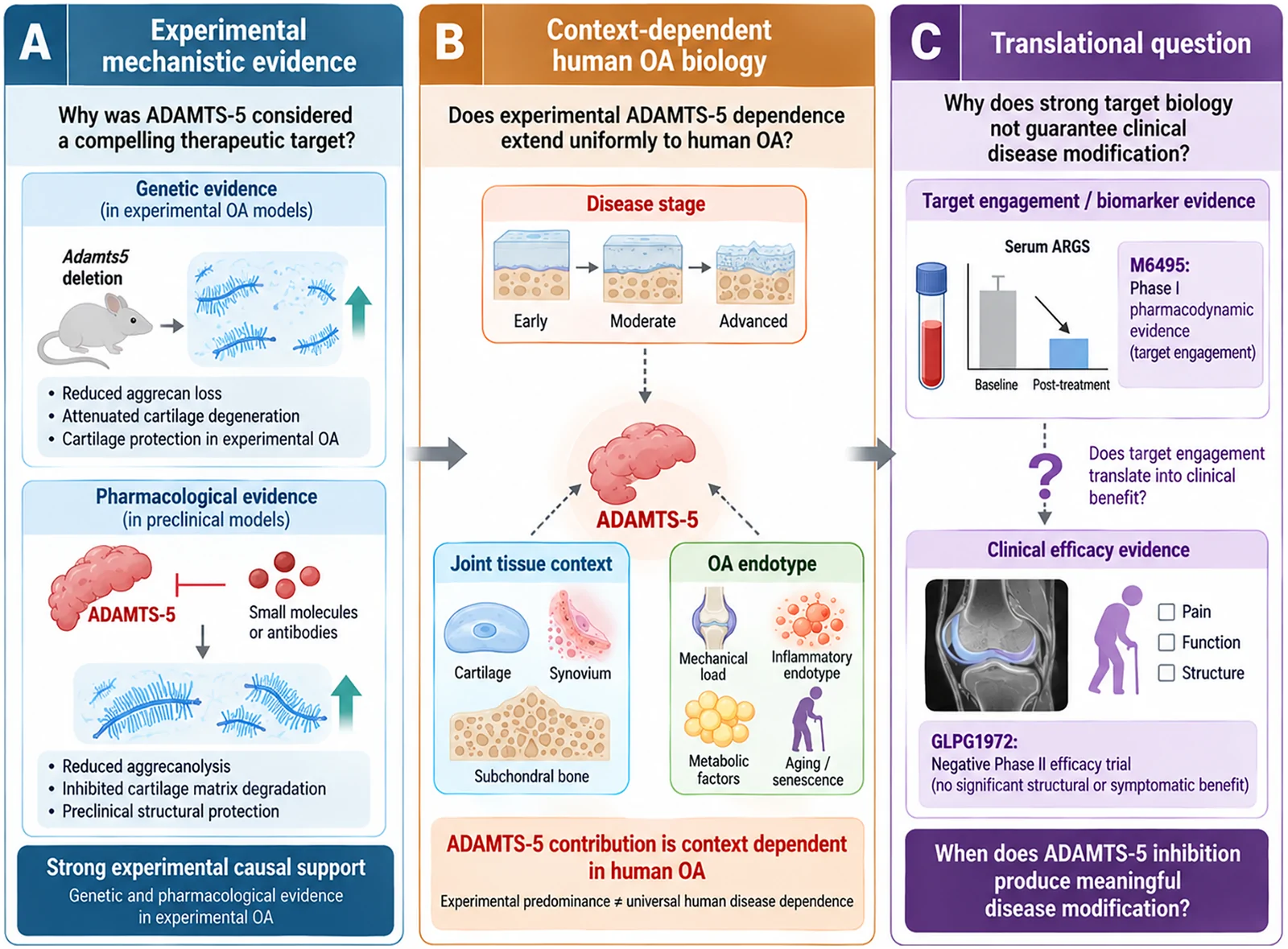
The ADAMTS-5 translational paradox in osteoarthritis: from experimental causality to uncertain clinical benefit. **(A)** Genetic deletion and pharmacological inhibition establish a major causal role for ADAMTS-5 in aggrecan degradation and cartilage damage in experimental osteoarthritis (OA). **(B)** In human OA, the contribution of ADAMTS-5 is context dependent, varying with disease stage, OA endotype, and joint tissue environment. **(C)** Clinical evidence highlights the distinction between pharmacodynamic target engagement and demonstrated disease modification: M6495 has shown ARGS biomarker reduction in Phase I studies, whereas GLPG1972/S201086 failed to demonstrate structural or symptomatic benefit in a Phase II trial. Together, these findings underscore that strong experimental target validation does not necessarily predict clinical efficacy in heterogeneous human OA.

However, despite strong preclinical evidence, clinical translation has been more complex (Figure 1). In the Phase II ROCCELLA trial, the selective ADAMTS-5 inhibitor GLPG1972/S201086 did not significantly reduce magnetic resonance imaging (MRI)-measured cartilage loss or improve symptomatic outcomes (19). By contrast, the available human data for the anti-ADAMTS-5 nanobody M6495 are predominantly Phase I safety, pharmacokinetic, and pharmacodynamic studies and should not be interpreted as a negative efficacy trial (20, 21). This distinction illustrates the central translational question addressed in this review: why does a target with strong experimental biology and measurable target engagement not necessarily produce disease modification in an unselected clinical population?

In addition to its pathological role in cartilage degradation, ADAMTS-5 also participates in normal physiological processes, including extracellular matrix remodeling, tissue repair, and neural homeostasis (22, 23). Disruption of ADAMTS-5 activity has been shown to impair tissue repair and alter ECM turnover, highlighting its functional importance beyond osteoarthritis (22). Furthermore, ADAMTS proteoglycanases are involved in central nervous system organization and synaptic plasticity, suggesting broader roles in tissue homeostasis (24). These physiological functions raise important safety concerns, as systemic or long-term inhibition of ADAMTS-5 may lead to unintended systemic or on-target physiological effects and disruption of normal tissue remodeling. Such considerations underscore the need for more refined therapeutic strategies, including spatially restricted delivery, temporal control of enzyme inhibition, and integration with broader disease-modifying approaches (25, 26).

The present focus on ADAMTS-5 does not imply that osteoarthritis is driven by this protease alone or that other inflammatory and matrix-degrading pathways are of lesser importance. Rather, ADAMTS-5 provides a particularly informative translational case study. In contrast to upstream pleiotropic mediators such as IL-1β and TNF-α, which regulate broad inflammatory and catabolic programs, ADAMTS-5 acts more proximally as a matrix-degrading effector through the cleavage of aggrecan. Its role is supported by strong genetic and pharmacological evidence in experimental OA, measurable aggrecan neoepitope biomarkers that permit pharmacodynamic assessment, and the development of selective inhibitors that have progressed into clinical testing, including a completed Phase II efficacy trial. At the same time, cytokines and other proteases, including IL-1β, TNF-α, ADAMTS-4, and MMP-13, interact with or compensate for ADAMTS-5 within the broader OA disease network. We therefore use ADAMTS-5 not as an isolated or uniquely dominant disease driver, but as a focused model for examining why strong mechanistic validation does not necessarily translate into clinically meaningful disease modification in a heterogeneous, stage-dependent, whole-joint disorder.

In this review, we use ADAMTS-5 as a focused translational model to critically examine the interface between molecular mechanism and therapeutic efficacy in OA. We first summarize its structural characteristics, regulation, and role in aggrecan degradation, and then evaluate current therapeutic strategies and the biological and clinical factors that may limit their translation. Particular emphasis is placed on disease stage, protease-network compensation, biomarker–outcome dissociation, patient heterogeneity, and spatially and temporally controlled intervention. By integrating mechanistic and clinical evidence, we aim to define a framework for the development of more precisely targeted ADAMTS-5-based strategies and, more broadly, for understanding why well-validated molecular targets may fail to produce consistent disease modification in OA. The overall framework is summarized in Figure 1, which positions ADAMTS-5 as a mechanistically well-supported but context-dependent matrix effector and highlights the key biological and translational factors that may uncouple target engagement from clinically meaningful disease modification.

## 2 Structural and functional characteristics of ADAMTS-5

ADAMTS-5 is a secreted zinc-dependent metalloproteinase of the ADAMTS (A Disintegrin and Metalloproteinase with Thrombospondin motifs) family. It is one of the principal aggrecanases implicated in cartilage ECM degradation and has particularly strong causal support from experimental OA models (9, 27). Its multidomain structure underlies both substrate recognition and catalytic efficiency (11).

### 2.1 Domain architecture and catalytic function

ADAMTS-5 is synthesized as a multidomain precursor comprising a signal peptide, propeptide, catalytic domain, disintegrin-like domain, thrombospondin type 1 repeat (TSR), cysteine-rich domain, and C-terminal TSR motifs (28, 29). Activation requires proteolytic removal of the propeptide by proprotein convertases such as furin, exposing the catalytic zinc-binding site that mediates substrate cleavage (11, 29).

The metalloproteinase domain contains the conserved HEXXHXXGXXH motif, which coordinates a catalytic Zn^2^⁺ ion essential for proteolysis 28. This domain is responsible for cleaving aggrecan at specific sites, most notably the Glu373–Ala374 bond within the interglobular domain (IGD) (30), a critical event that leads to rapid loss of aggrecan from the cartilage matrix (15). Importantly, this event occurs early in OA progression and precedes collagen network disruption, highlighting its role in initiating cartilage structural failure (28).

### 2.2 Exosite-mediated substrate recognition and specificity

Beyond the catalytic domain, ADAMTS-5 possesses multiple ancillary domains that contribute to substrate recognition through exosite interactions (27, 28). These exosites—located within the disintegrin-like, cysteine-rich, and TSR domains—facilitate binding to glycosaminoglycan-rich regions of aggrecan and enhance substrate affinity and cleavage efficiency (11).

This modular architecture enables ADAMTS-5 to achieve high specificity for large proteoglycan substrates such as aggrecan, versican, and brevican, distinguishing it from matrix metalloproteinases (MMPs), which primarily target collagenous components of the ECM (29, 31). Structural and biochemical studies have demonstrated that electrostatic complementarity, spatial configuration of the active site cleft, and non-covalent interactions such as sulfur–π stacking collectively contribute to precise substrate positioning and catalysis (11).

Importantly, exosite-mediated interactions are increasingly recognized as critical determinants of ADAMTS-5 function and represent promising targets for the development of selective inhibitors that avoid off-target effects associated with catalytic-site blockade (11, 18).

### 2.3 Experimental evidence for ADAMTS-5-mediated aggrecan degradation

Although both ADAMTS-4 and ADAMTS-5 exhibit aggrecanase activity, the strongest evidence for functional predominance of ADAMTS-5 comes from mouse cartilage and experimental OA models. ADAMTS-5-deficient mice are protected from cartilage erosion, whereas ADAMTS-4 knockout provides substantially less protection in these models (15, 32, 33). These experiments establish ADAMTS-5 as the major aggrecanase in mouse cartilage *in vivo*, but they do not prove uniform dominance across all human OA phenotypes or disease stages. Human cartilage studies support contributions from both ADAMTS-4 and ADAMTS-5, with relative importance likely varying according to tissue context and inflammatory state (14, 16, 28, 34, 35).

Furthermore, ADAMTS-5 activity is tightly regulated by its localization within the pericellular matrix, where it interacts with ECM components and cell surface molecules (36). This spatial regulation ensures that proteolytic activity is concentrated in regions of active cartilage remodeling but may also facilitate localized amplification of matrix degradation under pathological conditions (37). Together, these structural and functional features are linking the multidomain architecture of ADAMTS-5 to aggrecan recognition and cleavage while distinguishing the strong evidence for its predominance in experimental OA from the more context-dependent aggrecanase landscape in human disease.

### 2.4 Integration into cartilage catabolic networks

At the molecular level, ADAMTS-5 does not act in isolation but functions within a coordinated proteolytic and inflammatory network. Initial cleavage of aggrecan by ADAMTS-5 disrupts the structural integrity of the cartilage extracellular matrix, leading to a loss of compressive stiffness and biomechanical resilience (15). This early proteoglycan depletion exposes the collagen fibrillar network to subsequent degradation by matrix metalloproteinases (MMPs), particularly MMP-13, thereby initiating a sequential proteolytic cascade that drives irreversible matrix breakdown (38–40).

Furthermore, matrix fragments generated during ADAMTS-5-mediated aggrecanolysis can act as bioactive damage-associated molecular patterns (DAMPs), activating innate immune pathways and amplifying inflammatory signaling within the joint microenvironment (41).

Collectively, these findings indicate that ADAMTS-5 operates as a central node linking mechanical matrix disruption to inflammatory amplification, and suggest that targeting ADAMTS-5 alone may be insufficient to halt disease progression without addressing the broader degradative network.

## 3 ADAMTS-5 in osteoarthritis pathogenesis: a conceptual multi-stage framework

OA progression is a dynamic, multifactorial process driven by interactions among mechanical stress, inflammatory signaling, and ECM degradation. To organize these relationships, we use a three-stage conceptual framework—initiation, amplification, and chronic remodeling—to describe how ADAMTS-5 activity may be positioned across disease progression. These stages are not proposed as a fixed temporal sequence in every patient; they overlap, may recur, and are expected to differ among OA endotypes and joint tissues (16, 42).

### 3.1 Initiation phase: aggrecan depletion and biomechanical destabilization

In experimental OA, early loss of aggrecan from the cartilage ECM is a prominent event in which ADAMTS-5 has a major role (Figure 2A) (15, 16, 28). Aggrecan is essential for cartilage hydration and compressive resistance because its fixed negative charge retains water (3). Cleavage within the interglobular domain promotes proteoglycan loss, reduces osmotic swelling pressure, and weakens load-bearing capacity (11, 28). In human OA, however, the timing and relative contribution of ADAMTS-5-mediated aggrecanolysis are heterogeneous and should not be assumed to follow an identical sequence in every patient.

**Figure 2:**
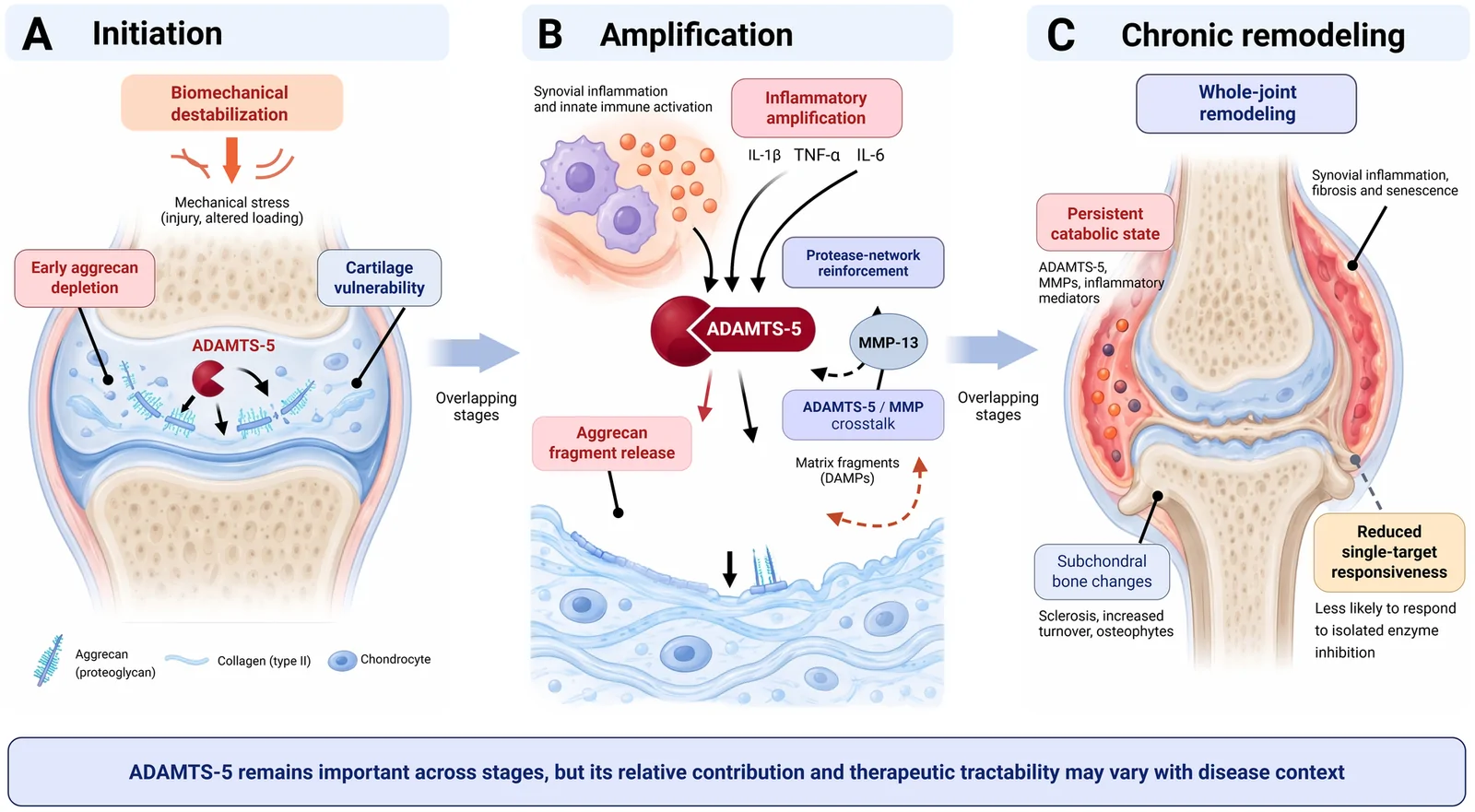
Conceptual multi-stage framework for ADAMTS-5-associated osteoarthritis progression. The proposed framework comprises overlapping rather than obligatory sequential stages. **(A)** During initiation, aggrecan depletion and matrix weakening increase cartilage susceptibility to mechanical injury. **(B)** During amplification, aggrecanolysis, matrix-derived danger signals, inflammatory signaling, and protease crosstalk reinforce the catabolic environment. **(C)** During chronic remodeling, persistent matrix loss becomes integrated with synovial inflammation, subchondral bone remodeling, cellular senescence, and other whole-joint changes, reducing the likelihood that isolated ADAMTS-5 inhibition will be sufficient for disease modification.

This biochemical disruption translates into biomechanical vulnerability, rendering cartilage more susceptible to mechanical stress and microdamage (Figure 2A). Importantly, this phase occurs prior to significant collagen network degradation, indicating that ADAMTS-5-driven aggrecanolysis is a key initiating step rather than a secondary consequence of cartilage breakdown (15, 28). Evidence from animal models further demonstrates that genetic ablation of ADAMTS-5 preserves aggrecan content and maintains cartilage integrity during early OA, underscoring its causal role in disease initiation (15, 33). This stage-dependent organization is illustrated in Figure 2 as a conceptual framework in which initiation, amplification, and chronic remodeling represent overlapping and potentially recurrent disease states rather than an obligatory linear sequence.

### 3.2 Amplification phase: protease crosstalk and inflammatory feedback loops

Following initial ECM disruption, ADAMTS-5 activity contributes to the establishment of a self-amplifying catabolic environment (Figure 2B). Loss of aggrecan alters cartilage matrix organization and facilitates subsequent collagen network damage by matrix metalloproteinases (43), particularly MMP-13, thereby amplifying matrix breakdown. In parallel, aggrecan fragments generated downstream of aggrecanolysis can act as damage-associated molecular patterns and activate Toll-like receptor signaling, especially Toll-like receptor2 (TLR2), on joint cells, triggering inflammatory cascades (41). These signals converge on NF-*κ*B and related pathways to promote cytokine production and further upregulate ADAMTS-5, forming a positive feedback loop (44, 45). In addition, crosstalk with Wnt/β-catenin and TGF-β/SMAD signaling further shifts chondrocytes toward catabolic and hypertrophic phenotypes, exacerbating disease progression (46–48).

### 3.3 Chronic remodeling phase: tissue adaptation and irreversible joint damage

As OA progresses, sustained ADAMTS-5 activity remains embedded within a chronic, whole-joint remodeling process (Figure 2C). Persistent extracellular matrix (ECM) degradation is accompanied by structural alterations not only in cartilage but also in subchondral bone and synovium, reflecting the complex and multi-tissue nature of the disease (42, 49). Increased mechanical loading on compromised cartilage further accelerates subchondral bone sclerosis and osteophyte formation, while ongoing low-grade inflammation promotes synovial hyperplasia and fibrosis (42, 49).

At this stage, the role of ADAMTS-5 becomes increasingly context-dependent. Although continued aggrecan degradation contributes to disease progression, ADAMTS-5 also participates in physiological ECM turnover and tissue repair processes. Experimental evidence has shown that deletion of ADAMTS-5 can impair normal tissue repair and disrupt matrix homeostasis, highlighting its essential role beyond pathological conditions (22, 28). This dual function complicates therapeutic strategies targeting ADAMTS-5, as complete or sustained inhibition may interfere with normal tissue remodeling and homeostasis. Moreover, chronic OA is characterized by cellular senescence, altered mechanotransduction, and persistent low-grade inflammation, all of which contribute to a self-perpetuating catabolic environment (42, 50). Mechanical overloading has been shown to promote chondrocyte senescence and accelerate OA progression, while inflammatory–senescence crosstalk further amplifies degenerative signaling pathways (50, 51). These factors may sustain catabolic signaling and maintain ADAMTS-5 expression beyond the initial triggers, ultimately rendering advanced disease less responsive to single-target therapeutic interventions.

Taken together, ADAMTS-5 can be viewed as a central node in OA pathogenesis, integrating early aggrecan degradation with broader biomechanical, biochemical, and inflammatory processes across disease progression (9, 19). Its role as a major aggrecanase makes it an attractive target for early intervention; however, emerging clinical and translational evidence indicates that inhibition of ADAMTS-5 alone may be insufficient to achieve consistent structural or symptomatic benefit (19, 20). This limitation likely reflects both the complexity of catabolic feedback networks in OA and the broader physiological roles of ADAMTS proteases in tissue remodeling and extracellular matrix turnover (52). In addition, the heterogeneous responses observed in clinical trials further underscore the stage-dependent and context-dependent role of ADAMTS-5 in disease progression (19, 20). Accordingly, these findings support a shift away from uniform systemic inhibition toward temporally and spatially controlled therapeutic strategies, including localized and precision-targeted interventions (9, 53).

## 4 Multi-layer regulation of ADAMTS-5 expression in osteoarthritis

The expression and activity of ADAMTS-5 are tightly controlled by a complex and multi-layered regulatory network that integrates inflammatory stimuli, intracellular signaling pathways, and epigenetic mechanisms (16, 44, 45). Pro-inflammatory cytokines, such as IL-1β and TNF-α, activate key transcriptional regulators including NF-*κ*B, which directly promotes ADAMTS-5 expression in chondrocytes during osteoarthritis (OA) progression (44). In parallel, non-coding RNAs, particularly microRNAs, further modulate ADAMTS-5 expression through post-transcriptional mechanisms, contributing to dynamic and context-dependent regulation (45, 54).

In addition to biochemical signaling, mechanical stress and alterations in tissue homeostasis play critical roles in shaping ADAMTS-5 activity. Mechanical loading has been shown to influence cartilage signaling pathways, including TGF-β activation, thereby modulating extracellular matrix turnover and chondrocyte phenotype (47). Furthermore, signaling pathways such as Wnt/β-catenin interact with inflammatory and mechanical cues to regulate catabolic responses in OA (46).

Collectively, this multi-layered regulatory architecture enables ADAMTS-5 to respond dynamically to changes in the joint microenvironment, integrating inflammatory, mechanical, and epigenetic signals across different stages of disease progression. Understanding this network is therefore essential for the development of therapeutic strategies that extend beyond direct enzyme inhibition and instead target upstream regulatory mechanisms and context-dependent pathways (16, 55). ADAMTS-5 expression emerges from the convergence of inflammatory, mechanical, intracellular, post-transcriptional, and systemic regulatory inputs rather than from a single linear signaling pathway.

### 4.1 Inflammatory cytokine-driven regulation

Inflammatory cytokines represent primary upstream drivers of ADAMTS-5 expression in osteoarthritic joints. Among these, interleukin-1β (IL-1β) and tumor necrosis factor-α (TNF-α) are among the most potent inducers reported in experimental and clinical OA settings (16). These cytokines activate canonical pro-inflammatory signaling pathways, particularly nuclear factor-*κ*B (NF-*κ*B), leading to nuclear translocation of the p65 (RelA) subunit and direct transcriptional activation of catabolic genes, including ADAMTS-5 (44).

In parallel, IL-1β and TNF-α stimulate mitogen-activated protein kinase (MAPK) cascades, including p38 and c-Jun N-terminal kinase (JNK), which enhance transcriptional activity through downstream effectors such as activator protein-1 (AP-1) and runt-related transcription factor 2 (Runx2) (40). These pathways collectively amplify ADAMTS-5 expression and promote matrix degradation.

Importantly, cytokine-mediated regulation is not unidirectional. Feedback mechanisms exist that partially buffer excessive catabolic activity. For example, IL-1β-induced expression of specific microRNAs, such as miR-30a, can suppress ADAMTS-5 expression at the post-transcriptional level, indicating the presence of intrinsic regulatory feedback loops (45). Additionally, cytokines such as interleukin-6 (IL-6) exhibit context-dependent roles, functioning as pro-inflammatory mediators during early disease stages while potentially exerting modulatory or protective effects in later phases (56).

### 4.2 Integration of intracellular signaling pathways

Beyond cytokine signaling, ADAMTS-5 expression is shaped by the convergence of multiple intracellular pathways that coordinate chondrocyte responses to environmental stimuli. The NF-*κ*B and MAPK pathways form the central axis of inflammatory signal transduction, jointly amplifying ADAMTS-5 transcription and sustaining a pro-catabolic transcriptional program (16, 44).

The transforming growth factor-β (TGF-β)/SMAD pathway exerts context-dependent regulation. Physiological TGF-β signaling contributes to cartilage homeostasis, whereas altered mechanical loading and dysregulated TGF-β/SMAD signaling in OA can shift chondrocytes toward catabolic or hypertrophic states. Mechanical stress can change the configuration of TGF-β activation, and experimental inhibition of SMAD3 has been reported to reduce ADAMTS-5 expression in early OA models (47, 48). These effects intersect with cartilage-regulatory microRNAs, including miR-140, rather than constituting a simple linear TGF-β→ADAMTS-5 pathway (57).

The Wnt/β-catenin pathway also plays a dual role. Basal Wnt signaling is required for cartilage maintenance, whereas excessive activation promotes chondrocyte hypertrophy and upregulation of catabolic enzymes, including ADAMTS-5 and matrix metalloproteinases (46). Conversely, excessive inhibition of Wnt signaling may impair cartilage integrity, underscoring the importance of maintaining signaling balance.

Similarly, the phosphatidylinositol 3-kinase (PI3 K)/Akt pathway demonstrates bidirectional regulation. Under inflammatory conditions, PI3 K/Akt signaling enhances ADAMTS-5 expression through downstream activation of NF-*κ*B, whereas under anabolic or homeostatic conditions, such as stimulation by insulin-like growth factor-1 (IGF-1), PI3 K/Akt promotes SOX9 activity, which suppresses ADAMTS-5 expression and supports extracellular matrix synthesis (58, 59).

### 4.3 Epigenetic and post-transcriptional regulation

In addition to transcriptional control, ADAMTS-5 expression is extensively regulated at the post-transcriptional and epigenetic levels, primarily through non-coding RNAs. MicroRNAs (miRNAs) serve as key negative regulators of ADAMTS-5 by binding to the 3′ untranslated region (3′-UTR) of its mRNA, resulting in translational repression or mRNA degradation (16).

Several miRNAs, including miR-140, miR-27a-3p, miR-30a, miR-132, and miR-137, have been reported to regulate ADAMTS-5 directly or indirectly (45, 57, 60, 61). These RNAs are not ADAMTS-5-selective pharmacological switches. miR-140, for example, is a broader cartilage-homeostatic regulator, while inflammatory miRNA programs can simultaneously influence MMP expression, SOX9-dependent matrix synthesis and differentiation, apoptosis, and NF-*κ*B/MAPK signaling. Consequently, manipulating an miRNA can alter the balance of anabolic, catabolic, and inflammatory pathways in addition to changing ADAMTS-5 expression.

Long non-coding RNAs (lncRNAs) further modulate this network through competing-endogenous-RNA and related mechanisms. Representative examples illustrate this pleiotropy: the XIST/miR-1277-5p axis can co-regulate ADAMTS-5 and MMP-13; PVT1/miR-140 is linked to broader extracellular-matrix degradation; and MALAT1/miR-145 influences chondrocyte viability and cartilage-matrix components in addition to ADAMTS-5 (62–64). MIR22HG- and XIST-related axes further connect ADAMTS-5 regulation with OA progression and chondrogenic differentiation (65, 66). Thus, RNA-based interventions should be interpreted as network perturbations whose therapeutic effects cannot be attributed solely to ADAMTS-5 suppression. Such pleiotropy may be advantageous when multiple catabolic pathways are co-modulated, but it also increases the need for cell-selective delivery, dose control, and pathway-level safety assessment.

Emerging evidence also suggests that chromatin remodeling and histone modifications may influence ADAMTS-5 transcription; however, these mechanisms remain less well characterized in osteoarthritis and require further investigation (67). Representative lncRNA–miRNA regulatory axes linked to ADAMTS-5, together with their broader effects on cartilage homeostasis and OA-associated catabolic phenotypes, are summarized in Table 1.

**Table 1:** LncRNA–miRNA–ADAMTS-5 axes implicated in osteoarthritis progression.

LncRNA	Interacting miRNA	OA context/ model	Main phenotype/ downstream effect	Optimized mechanism summary	Reference
MIR22HG	miR-9-3p	Human OA cartilage/chondrocytes; IL-1β-treated chondrocytes	↑ ECM degradation; ↑ chondrocyte apoptosis; OA progression	MIR22HG acts as a ceRNA for miR-9-3p, relieving miR-9-3p-mediated repression of ADAMTS-5 and thereby promoting matrix-degrading activity under inflammatory conditions.	(65)
MALAT1	miR-145	Human OA chondrocytes; IL-1β-induced *in vitro* model	↓ viability; ↑ matrix degradation; OA-like catabolic phenotype	MALAT1 sponges miR-145, releases ADAMTS-5 from miR-145-mediated suppression, and aggravates IL-1β-induced cartilage matrix breakdown.	(62)
XIST	miR-1277-5p	Human OA cartilage/ chondrocytes; IL-1β-treated chondrocytes; rat OA model	↑ ECM degradation; ↑ MMP-13; *in vivo* cartilage damage	XIST functions as a ceRNA of miR-1277-5p, derepressing ADAMTS-5 (and MMP-13) and promoting extracellular matrix degradation in OA chondrocytes and *in vivo*.	(63)
	miR-27b-3p	Temporomandibular joint OA; synovium-derived mesenchymal stem cells	↓ chondrogenic differentiation; impaired cartilage-repair phenotype	XIST sponges miR-27b-3p, increases ADAMTS-5 expression, and suppresses chondrogenic differentiation of synovium-derived mesenchymal stem cells in TMJ-OA.	(66)
PVT1	miR-140	Human OA chondrocytes; OA tissue/inflammatory *in vitro* model	↑ ECM degradation; ↑ MMP-13; worsened catabolic phenotype	PVT1 promotes OA-associated extracellular matrix degradation by acting as a ceRNA of miR-140, thereby increasing ADAMTS-5 and related catabolic responses.	(64)

ADAMTS-5, a disintegrin and metalloproteinase with thrombospondin motifs 5; ceRNA, competing endogenous RNA; ECM, extracellular matrix; IL-1β, interleukin-1 beta; lncRNA, long non-coding RNA; miRNA, microRNA; MMP-13, matrix metalloproteinase 13; OA, osteoarthritis; TMJ, temporomandibular joint.

### 4.4 A dynamic and context-dependent regulatory network

Collectively, the regulation of ADAMTS-5 is best understood as a dynamic, multi-layered network in which inflammatory signals, intracellular pathways, and epigenetic regulators interact to determine its expression and activity (16, 68). This regulatory architecture enables ADAMTS-5 to respond in a context-dependent manner to variations in mechanical loading, cytokine exposure, and tissue homeostasis.

Importantly, this complexity provides a mechanistic explanation for the limited efficacy of therapeutic strategies that target ADAMTS-5 alone. Rather than functioning as an isolated effector, ADAMTS-5 operates within an interconnected signaling network, suggesting that effective disease-modifying approaches may require modulation of upstream regulatory nodes or combinatorial targeting of multiple pathways (68).

### 4.5 The gut–joint axis as an upstream modifier of catabolic signaling

Emerging evidence implicates the gut microbiome as an upstream modifier of the inflammatory and metabolic environment in OA. Human cohort data have linked greater intestinal *Streptococcus* abundance to OA-related knee pain and joint effusion, supporting an association between microbial composition and local joint inflammation (69). Mechanistically, gut-barrier dysfunction may increase systemic exposure to microbial products such as lipopolysaccharide (LPS), which amplifies low-grade inflammation through LBP/CD14–TLR signaling, thereby reinforcing low-grade inflammatory and catabolic signaling; NF-*κ*B provides one mechanistic link to ADAMTS-5 induction in chondrocytes (44, 70). Consistent with this indirect gut–joint axis, oral administration of *γ*-aminobutyric acid (GABA)-producing Streptococcus thermophilus and Lactobacillus pentosus reduced ADAMTS-5 and other cartilage-catabolic markers in experimental OA (71). However, a direct microbiome-to-ADAMTS-5 causal pathway has not been established in patients. Dietary, probiotic, or other microbiome-directed interventions should therefore be considered potential upstream modifiers of the inflammatory–catabolic milieu rather than specific ADAMTS-5 inhibitors.

## 5 Therapeutic targeting of ADAMTS-5: From molecular inhibition to precision intervention

Despite strong mechanistic rationale, targeting ADAMTS-5 alone has not consistently translated into meaningful clinical benefit, highlighting the need to re-evaluate current therapeutic paradigms (9, 19). Over the past decade, multiple strategies have been developed to inhibit ADAMTS-5 activity or suppress its expression. Although these approaches have demonstrated promising effects in preclinical models, clinical translation has been limited (9, 17, 19). This section provides a critical overview of current therapeutic strategies, emphasizing both their mechanistic basis and their translational challenges. The current therapeutic landscape is organized in Figure 3 according to level of evidence and mechanism of intervention, distinguishing human clinical evidence from preclinical target-modulation strategies and delivery-enabling technologies.

**Figure 3:**
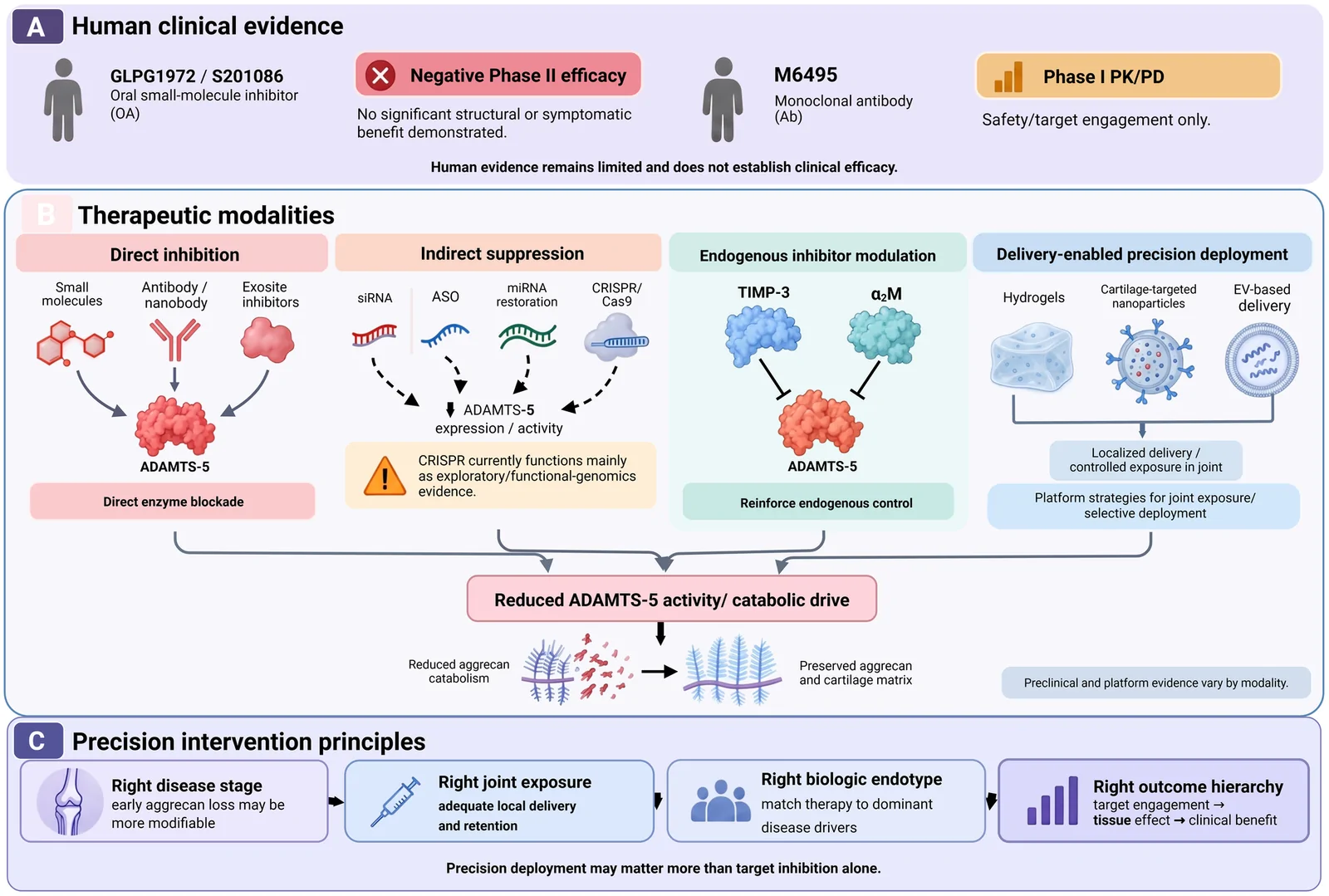
Therapeutic strategies targeting ADAMTS-5: from direct inhibition to precision intervention. **(A)** Human clinical evidence differs substantially by development stage: GLPG1972/S201086 failed to demonstrate significant structural or symptomatic benefit in the Phase II ROCCELLA trial, whereas M6495 has primarily shown Phase I safety, pharmacokinetic, pharmacodynamic, and target-engagement evidence without definitive efficacy testing. **(B)** Therapeutic approaches include direct enzymatic inhibition, RNA-based suppression, modulation of endogenous inhibitors, exploratory genome-editing strategies, and delivery platforms such as hydrogels, nanoparticles, and extracellular vesicles. **(C)** Future development should align target modulation with disease stage, molecular endotype, local joint exposure, pharmacodynamic evidence, and clinically meaningful structural and symptomatic outcomes.

### 5.1 Direct inhibition of ADAMTS-5 activity

Direct targeting of ADAMTS-5 has included small-molecule catalytic inhibitors, monoclonal antibodies/nanobodies, and mechanism-based exosite inhibitors. These modalities should be interpreted according to their distinct levels of clinical evidence rather than grouped as equivalent efficacy-stage programs.

GLPG1972/S201086 is a selective small-molecule ADAMTS-5 inhibitor with robust preclinical activity (17, 72). In the 52-week, 932-participant Phase II ROCCELLA trial, however, none of the tested doses significantly reduced central medial femorotibial cartilage-thickness loss versus placebo, and no significant benefit was observed for secondary structural or symptomatic endpoints (19). This constitutes a negative efficacy-stage trial and provides the clearest clinical example of the translational gap discussed in this review.

M6495 should be interpreted differently. Two randomized Phase I studies in healthy volunteers and patients with OA primarily evaluated safety, pharmacokinetics, and pharmacodynamics. M6495 was generally well tolerated and produced dose-dependent reductions in serum ARGS, supporting systemic target engagement (20, 21). These studies were not designed or powered to establish structural disease modification or symptomatic efficacy; therefore, the current M6495 evidence represents early-phase safety and pharmacodynamic proof-of-mechanism rather than an efficacy failure.

An emerging strategy involves targeting exosites rather than the catalytic domain. By disrupting substrate recognition without fully abolishing enzymatic activity, exosite inhibitors may achieve greater selectivity while preserving physiological functions of ADAMTS-5 (11). Although this approach represents a promising direction for next-generation therapeutics, it remains largely in the preclinical stage.

### 5.2 Indirect suppression via gene and RNA-based approaches

Given the limitations of direct inhibition, alternative strategies have focused on suppressing ADAMTS-5 expression at the transcriptional or post-transcriptional level. RNA-based therapies, including small interfering RNA (siRNA), antisense oligonucleotides (ASOs), and microRNA (miRNA) mimics, have shown considerable potential in preclinical models (54). Among these, miR-140 has received particular attention due to its essential role in maintaining cartilage homeostasis and its ability to directly repress ADAMTS-5 expression (57). Restoration of miR-140 levels has been associated with reduced extracellular matrix degradation and improved cartilage integrity in experimental models.

CRISPR/Cas9 has also been used as a functional-genomics tool in primary human chondrocytes. The cited study used efficient CRISPR editing to identify mechanosensitive miR-140 targets and regulatory circuitry rather than to demonstrate therapeutic, long-term suppression of ADAMTS-5 in OA (73). Genome editing may eventually enable durable manipulation of catabolic pathways, but ADAMTS-5-directed CRISPR therapy remains conceptual and faces substantial challenges in delivery, tissue specificity, off-target editing, and regulatory evaluation.

Importantly, RNA-based approaches may provide a more physiologically compatible strategy by enabling partial and context-dependent suppression of ADAMTS-5 rather than complete enzymatic blockade, which may be advantageous given its roles in normal tissue homeostasis.

### 5.3 Modulation of endogenous inhibitors

Enhancing endogenous inhibitors of ADAMTS-5 represents an alternative therapeutic strategy. Tissue inhibitor of metalloproteinases-3 (TIMP-3) is the principal endogenous inhibitor of ADAMTS-5 and plays a critical role in maintaining extracellular matrix homeostasis (74). Reduced TIMP-3 activity has been associated with increased ADAMTS-5 activity and cartilage degradation in osteoarthritis.

Strategies to restore TIMP-3 function include gene therapy, recombinant protein delivery, and the development of engineered variants with enhanced specificity and stability (74). Similarly, *α*2-macroglobulin (*α*2M) has been investigated as a natural inhibitor capable of sequestering ADAMTS-5 through a molecular trapping mechanism (30). However, limited diffusion within cartilage and short intra-articular residence time may restrict its therapeutic efficacy.

Overall, targeting endogenous regulatory systems offers a more balanced approach to modulating ADAMTS-5 activity but requires optimized delivery strategies to achieve sustained therapeutic effects.

### 5.4 Advanced intra-articular delivery systems

A major challenge in targeting ADAMTS-5 is achieving effective and sustained drug concentrations within the joint while minimizing systemic exposure. To address this, advanced intra-articular delivery systems have been developed (53).

Hydrogel-based systems provide biocompatible matrices for sustained release of therapeutic agents, enabling prolonged retention within the joint cavity (75, 76). These systems can be engineered to respond to environmental cues such as pH or enzymatic activity, allowing controlled drug release in diseased tissues. Nanoparticle-based delivery platforms improve drug stability, enhance cellular uptake, and enable targeted delivery to cartilage through ligand functionalization (26, 77). These features significantly improve drug retention and specificity within the joint microenvironment. Extracellular vesicles (EVs), particularly those derived from mesenchymal stem cells, have emerged as promising delivery vehicles due to their inherent biocompatibility and ability to transport bioactive molecules (78, 79). EVs can modulate ADAMTS-5 expression indirectly through the transfer of regulatory RNAs and proteins, while also exerting immunomodulatory effects.

Collectively, these delivery strategies are essential for overcoming key barriers to clinical translation, including poor bioavailability, rapid clearance, and lack of tissue specificity. The major preclinical and enabling strategies discussed above, including exosite inhibition, RNA-based modulation, endogenous inhibitor restoration, and localized delivery platforms, are compared in Table 2 with respect to mechanism, development stage, and translational implications.

**Table 2:** Preclinical and platform strategies relevant to ADAMTS-5 targeting in osteoarthritis.

Therapeutic agent/strategy	Modality	Primary mechanism relevant to ADAMTS-5	Current research stage	Key findings/translational interpretation	Reference
Exosite inhibitors/substrate-recognition blockers	Mechanism-based small molecules/glycoconjugates	Target ancillary substrate-recognition regions rather than the catalytic zinc-binding site, aiming to improve selectivity and preserve physiological turnover	Preclinical	Exosite-directed strategies are supported by structural and substrate-recognition studies and remain an attractive next-generation direction, but no mature clinical OA program is yet established.	(11,13,86)
siRNA targeting ADAMTS-5	RNA interference	Post-transcriptional silencing of ADAMTS-5 mRNA	Preclinical animal studies	Lentivirus-mediated siRNA against ADAMTS-5 reduced cartilage degradation in rat OA models, supporting gene-silencing approaches as a local disease-modifying strategy.	(87)
miR-140 replacement/mimic strategy	microRNA-based therapy	Restores cartilage-homeostatic miR-140, which represses ADAMTS-5 and other catabolic pathways	Preclinical/proof-of-concept	miR-140 is a cartilage-homeostatic regulator and a leading RNA candidate for indirect suppression of ADAMTS-5; translational challenges include delivery efficiency and durability.	(54,57)
CRISPR/Cas9-based functional genomics of ADAMTS-5-related regulatory programs	Genome editing/functional genomics	Identifies mechanosensitive and catabolic regulatory circuitry relevant to ADAMTS-5 expression	Exploratory/mechanistic	CRISPR editing has been used to define miR-140 regulatory targets in primary human chondrocytes; this is mechanistic evidence, not a therapeutic demonstration of durable ADAMTS-5 suppression.	(73)
TIMP-3 restoration/engineered TIMP-3 variants	Endogenous inhibitor modulation	Restores the principal endogenous inhibitor of ADAMTS-5 to rebalance matrix turnover	Preclinical	Aggrecanase-selective TIMP-3 variants protected articular cartilage *in vivo*, supporting endogenous-inhibitor enhancement as a biologically balanced alternative to complete enzyme blockade.	(74)
*α*2-Macroglobulin (α2M)	Endogenous inhibitor/biologic	Molecular trapping and sequestration of ADAMTS-4/5 proteases	Preclinical/mechanistic	Acts as an endogenous inhibitor of aggrecanases, but therapeutic translation is constrained by diffusion and residence-time limitations within the joint environment.	(30)
Hydrogel-based intra-articular delivery	Localized delivery platform	Prolonged local retention and controlled release of ADAMTS-5-directed agents	Preclinical/platform technology	Hydrogels can improve residence time and controlled release of small molecules, proteins, or nucleic acids in the joint; they are enabling technologies rather than direct ADAMTS-5 inhibitors *per se*.	(75,76)
Cartilage-targeted nanoparticles	Localized delivery platform	Enhance stability, penetration, and cartilage targeting of ADAMTS-5-directed payloads	Preclinical/platform technology	Nanocarriers improve local exposure and targeting specificity and are especially relevant for precision deployment of ADAMTS-5 inhibitors or RNA therapeutics.	(26,77)
Extracellular vesicle (EV)-based delivery	Biologic delivery platform	Transfers regulatory RNAs/proteins that can indirectly suppress ADAMTS-5 and modulate inflammation	Preclinical/emerging translational platform	EVs, particularly MSC-derived EVs, provide biocompatible local delivery and immunomodulation; they are promising for indirect ADAMTS-5 regulation, but standardization and payload control remain barriers.	(78,79)

ADAMTS-5, a disintegrin and metalloproteinase with thrombospondin motifs 5; α2M, alpha-2-macroglobulin; CRISPR/Cas9, clustered regularly interspaced short palindromic repeats/CRISPR-associated protein 9; ECM, extracellular matrix; EV, extracellular vesicle; miRNA, microRNA; MSC, mesenchymal stem cell; OA, osteoarthritis; siRNA, small interfering RNA; TIMP-3, tissue inhibitor of metalloproteinases 3.

### 5.5 Translational design principles

The therapeutic lesson is not simply to inhibit ADAMTS-5 more strongly, but to align target biology with disease stage, tissue exposure, and patient selection. An ADAMTS-5-directed strategy is most biologically plausible when active aggrecanolysis remains measurable and potentially reversible; later disease is more likely to require interventions that also address collagen damage, synovitis, subchondral bone remodeling, or repair failure. Local and sustained intra-articular delivery may improve joint exposure while limiting systemic inhibition, whereas combination therapy should be guided by evidence of residual pathway activity rather than used empirically (26, 53, 77, 78).

## 6 Translational barriers and safety challenges in targeting ADAMTS-5

Despite compelling mechanistic rationale and robust preclinical evidence, ADAMTS-5-directed therapies have not yet demonstrated consistent efficacy-stage clinical success. Importantly, the evidence is not uniform across agents: GLPG1972/S201086 has a negative Phase II efficacy trial, whereas M6495 currently has Phase I safety and target-engagement data without an efficacy verdict. This distinction is central to interpreting the translational barriers discussed below (19, 20). These translational challenges are integrated in Figure 4 as a sequence of pharmacological and biological thresholds linking joint exposure and target engagement to biological dependence, structural modification, symptomatic benefit, and ultimately durable disease modification.

**Figure 4:**
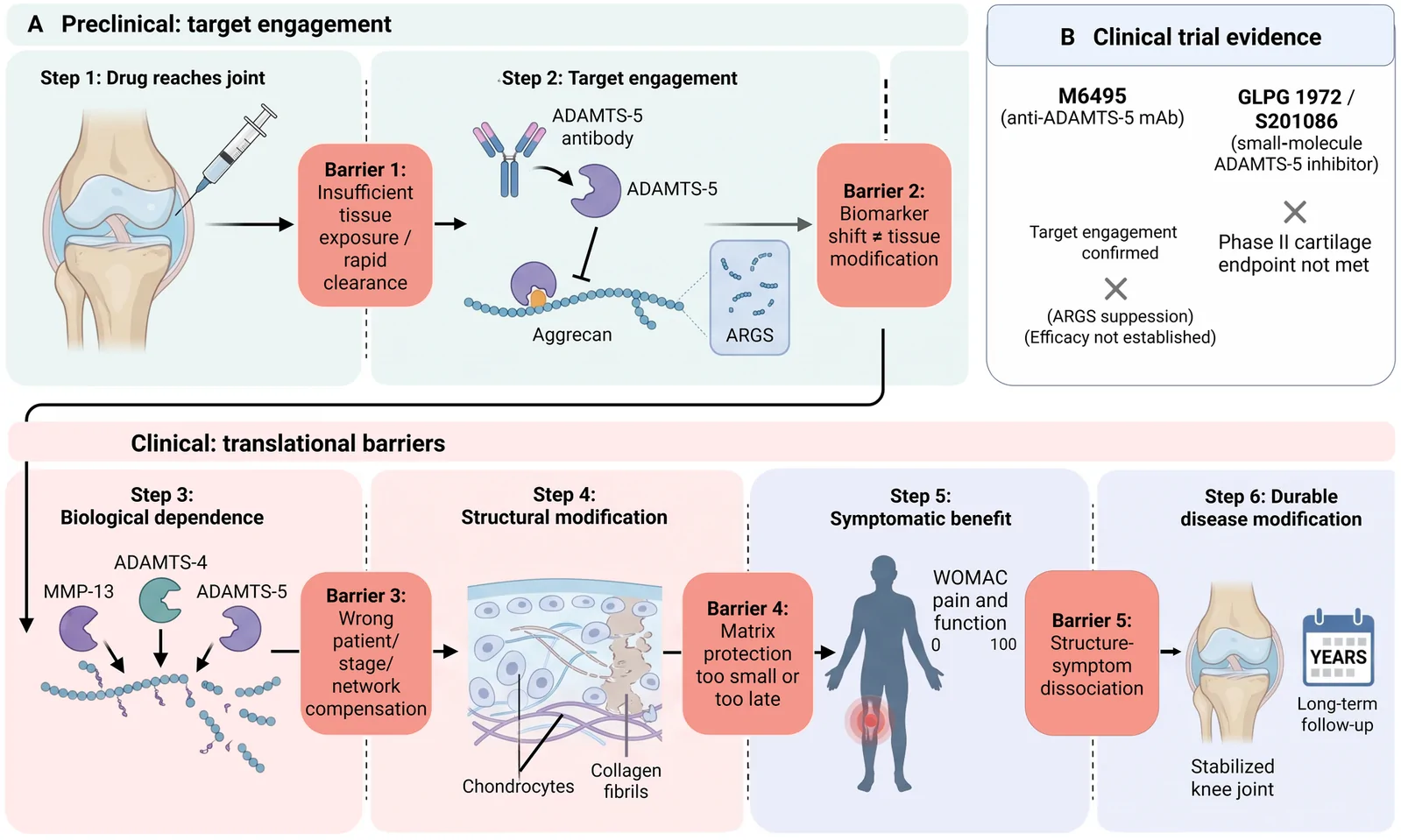
Translational barriers between ADAMTS-5 target engagement and clinical disease modification. **(A)** Translation from drug administration to durable clinical benefit requires sequential progression through six thresholds: adequate joint exposure, ADAMTS-5 target engagement, biological dependence on ongoing aggrecanolysis, structural modification, symptomatic benefit, and durable disease modification. Distinct barriers can interrupt this pathway at each stage, including insufficient tissue exposure, dissociation between biomarker modulation and tissue-level efficacy, inappropriate patient or disease-stage selection, protease-network compensation, inadequate or late structural rescue, and structure–symptom dissociation. Collectively, these barriers illustrate why pharmacodynamic target engagement is necessary but not sufficient for clinical disease modification. **(B)** Human clinical examples highlight this distinction: M6495 has demonstrated Phase I safety, pharmacokinetic/pharmacodynamic activity, and ADAMTS-5 target engagement without established efficacy, whereas GLPG1972/S201086 did not demonstrate structural or symptomatic efficacy in the Phase II ROCCELLA trial.

### 6.1 Disconnect between biomarker modulation and clinical outcomes

A central translational issue is that biochemical target engagement is not equivalent to clinical efficacy. Early human studies of ADAMTS-5 inhibitors have demonstrated modulation of aggrecan-fragment biomarkers such as ARGS (20, 21). For GLPG1972/S201086, this pharmacological rationale did not translate into structural or symptomatic benefit in ROCCELLA (19). For M6495, ARGS reduction establishes pharmacodynamic activity, but the available Phase I studies do not permit a conclusion about clinical efficacy. The appropriate inference is therefore agent- and phase-specific: target engagement has been shown, but disease modification has not been established.

This disconnect suggests that aggrecan degradation, while critical in early disease stages, may not be the primary driver of symptoms in established OA. Pain in OA is influenced by multiple pathological processes, including synovial inflammation, subchondral bone remodeling, and peripheral and central sensitization, which are not directly addressed by ADAMTS-5 inhibition (42). Accordingly, targeting a single molecular pathway may be insufficient to produce clinically meaningful outcomes, particularly in heterogeneous patient populations. The available human evidence is summarized separately in Table 3, emphasizing the distinction between the negative Phase II efficacy findings for GLPG1972/S201086 and the Phase I safety, pharmacokinetic, pharmacodynamic, and target-engagement evidence available for M6495.

**Table 3:** Human clinical evidence for direct ADAMTS-5 inhibitors in osteoarthritis.

Agent	Study phase/population	Target-engagement biomarker	Structural outcome	Symptomatic outcome	Safety/tolerability	Interpretation
GLPG1972/ S201086 (19,88)	Phase II ROCCELLA; 932 adults with symptomatic knee OA; 52 weeks	Earlier Phase I studies showed ARGS reduction; Phase II evaluated efficacy	No significant reduction in cMFTC cartilage-thickness loss or other structural secondary endpoints versus placebo	No significant benefit in WOMAC/pain or other clinical secondary endpoints	Treatment-emergent adverse events were broadly similar across groups	Negative efficacy-stage trial despite strong mechanistic rationale
M6495 (20,21)	Two Phase I randomized placebo-controlled studies; 54 healthy volunteers and 32 patients with OA	Dose-dependent reduction in serum ARGS supported ADAMTS-5 target engagement	Not established; Phase I studies were not designed/powered for structural efficacy	Not established; no efficacy conclusion should be drawn from Phase I studies	Generally well tolerated in the reported studies	Safety/PK/PD and target-engagement evidence only; efficacy remains to be tested

ADAMTS-5, a disintegrin and metalloproteinase with thrombospondin motifs 5; ARGS, aggrecan neoepitope generated by aggrecanase cleavage; cMFTC, central medial femorotibial compartment; OA, osteoarthritis; PD, pharmacodynamics; PK, pharmacokinetics; WOMAC, Western Ontario and McMaster Universities Osteoarthritis Index.

### 6.2 Disease heterogeneity and stage-dependent effects

Osteoarthritis is increasingly recognized as a heterogeneous disease comprising multiple phenotypes, including inflammatory, metabolic, mechanically driven, and aging-associated subtypes (42). The contribution of ADAMTS-5 varies across these phenotypes and across disease stages.

In experimental early OA, ADAMTS-5-mediated aggrecanolysis is prominent and therefore provides a biologically plausible window for intervention (15, 32, 80). In human OA, however, this window has not been prospectively defined. Once collagen-network damage, subchondral bone remodeling, synovitis, fibrosis, and pain sensitization are established, suppressing aggrecanase activity alone is unlikely to reverse the full disease state (9, 42).

Furthermore, the lack of molecular or phenotypic stratification in clinical trials may dilute therapeutic effects, as only a subset of patients—particularly those with active aggrecan degradation—may derive meaningful benefit from ADAMTS-5-targeted therapies.

### 6.3 Protease redundancy and compensatory mechanisms

Another major challenge arises from functional redundancy within the protease network. Although ADAMTS-5 is a major aggrecanase, particularly in experimental OA, other proteases, including ADAMTS-4 and matrix metalloproteinases (MMPs), also contribute to extracellular matrix degradation and may sustain catabolic activity in the joint (14, 28).

As a result, inhibition of ADAMTS-5 alone may not fully suppress matrix degradation, particularly as downstream processes such as collagen breakdown by MMP-13 continue to drive structural deterioration. This redundancy underscores the limitations of single-target approaches and supports the rationale for combination therapies that simultaneously target multiple components of the degradative network.

### 6.4 Physiological roles and safety concerns of ADAMTS-5 inhibition

Beyond its pathological role in OA, ADAMTS-5 and related proteoglycanases are involved in physiological processes including tissue development, extracellular matrix remodeling, and repair (9, 52). Experimental studies have demonstrated that loss of ADAMTS-5 activity may impair tissue repair processes, highlighting its role in maintaining tissue homeostasis (22).

In addition, ADAMTS family members have been implicated in diverse biological systems, including cardiovascular matrix remodeling, reproductive tissue biology, and central nervous system homeostasis (23, 52). Consequently, long-term or systemic inhibition of ADAMTS-5 may carry risks of unintended effects, such as impaired extracellular matrix turnover or altered tissue function.

Although the clinical relevance of these risks remains incompletely defined, these findings emphasize the importance of selectively targeting pathological activity while preserving physiological function.

### 6.5 Patient selection: From clinical phenotyping to molecular endotyping

For ADAMTS-5-directed therapy, clinical phenotyping alone is unlikely to be sufficiently specific. Age, pain pattern, radiographic grade, obesity, and inflammatory features remain useful for eligibility and safety, but they do not directly establish that ongoing aggrecanolysis is a dominant process. A more testable strategy is hierarchical enrichment: first define clinical stage and joint phenotype; then add molecular endotyping using aggrecan/cartilage-turnover biomarkers and quantitative imaging. Aggrecan-derived ARGS neoepitopes are particularly relevant because they directly report aggrecan cleavage and have shown pharmacodynamic responsiveness in human ADAMTS-5 inhibitor studies (20, 21, 81).

Single-cell transcriptomics can further resolve disease-relevant chondrocyte states, spatial profiling can preserve the anatomical context of catabolic programs across joint compartments, and interpretable machine-learning approaches may integrate molecular, imaging, and clinical variables to define reproducible OA subgroups (82–85). These approaches should currently be regarded as investigational stratification tools rather than validated companion diagnostics. Prospective studies are required to determine whether an ADAMTS-5-high or aggrecanase-active endotype predicts treatment response more effectively than clinical phenotyping alone.

### 6.6 A testable translational roadmap

Future trials should separate three questions: (i) is the drug reaching and inhibiting ADAMTS-5 in the joint; (ii) is the enrolled disease state biologically dependent on ongoing aggrecanolysis; and (iii) does this produce a meaningful structural or symptomatic benefit? A biomarker-enriched, earlier-stage population combined with quantitative MRI and predefined pharmacodynamic thresholds would directly test this sequence. Local delivery and rational combination therapy should be introduced when exposure or network compensation, rather than target engagement itself, is the limiting factor.

## 7 Conclusion and future perspectives

ADAMTS-5 remains one of the best-supported matrix-degrading targets in experimental OA, but its clinical translation illustrates the distinction between mechanistic centrality, pharmacodynamic target engagement, and demonstrated disease modification. Mouse genetics establish a major role in aggrecan degradation, whereas human OA is heterogeneous and multi-tissue. The negative Phase II ROCCELLA result therefore does not invalidate ADAMTS-5 biology, but it shows that selective enzyme inhibition alone was insufficient in the studied symptomatic knee-OA population. M6495, by contrast, currently provides early-phase safety and target-engagement evidence rather than an efficacy verdict.

The next generation of studies should be designed around this distinction. Earlier intervention, molecular endotyping for active aggrecanolysis, quantitative imaging, and local exposure control offer a more rigorous way to test whether a responsive ADAMTS-5-dependent state exists. Single-cell and spatial approaches can refine the relevant tissue and cell states, while data-driven integration may improve trial enrichment. Combination therapy should be reserved for biologically justified settings in which inflammatory, bone, or repair pathways remain active despite aggrecanase inhibition. In this framework, ADAMTS-5 is best viewed as a context-dependent therapeutic node whose clinical value must be demonstrated in the right patient, tissue, and disease stage.
